# 

**Published:** 1903-11-21

**Authors:** 


### Publications.

JUST ISSUED EIGHTH EDITION. Crown 8vo, 10s. 6d.

### PHARMACY, MATERIA MEDICA AND THERAPEUTICS

Is now rewritten and printed in large type, and is made a companion to the following volume. It contains an account of the action **of every drug in the B.P.**, as well as a section upon all the newest drugs, with an Introduction to the Art of Dispensing. With woodcuts, prescriptions, &c.

Also FOURTH EDITION. 1055 pages, 8vo. 16s.

### DICTIONARY OF TREATMENT.

Containing a Revised and up-to-date Article upon every Diseased Condition, whether Medical, Surgical, Gynæcological, or Ophthalmological, as well as Articles upon Poisoning, Drowning, and the various Surgical Emergencies and Accidents, arranged for Rapid Reference.

By W. WHITLA, M.A., M.D., Professor of Materia Medica, Queen's College, Belfast; Senior Physician to the Royal Victoria Hospital, &c.

The Publisher will supply both volumes, carriage paid, for 21/-

London: HENRY RENSHAW, 356 Strand.

---

2nd Edition. Revised and Enlarged. Now Ready.
Post 8vo. pp. xv.-268, and 39 Illustrations in the text.
Cash price 4/6, postage 4d.

### PRACTICAL TEXT-BOOK OF MIDWIFERY FOR NURSES.

By ROBERT JARDINE, M.D. Edin., M.R.C.S. Eng., F.F.P. and S. Glas.
Professor of Midwifery in St. Mungo's College, Glasgow; Physician to the Glasgow Maternity Hospital; late President of the Glasgow Obstetrical and Gynæcological Society.

"It is the best suited for its purpose of any book of the kind which we have come across . . . For nurses the little book is an improvement on any similar book of the kind with which we are familiar."
*The Dublin Journal of Medical Science.*

W. F. CLAY, 18 Teviot Place, Edinburgh.

---

Crown 8vo. Illustrated. Price 2s. 6d. net.

### ANÆSTHETICS. By J. BLUMFELD,

M.D.Cantab., Senior Anæsthetist to St. George's Hospital; Anæsthetist to the Grosvenor Hospital for Women and Children, London, and to the National Dental and Metropolitan Hospitals.

*Lancet.*—"....In writing this handbook the author has been quite successful." *Australasian Medical Gazette.*—"We can strongly commend this book." *Medical Chronicle.*—"The book is one which we can heartily recommend to senior students and practitioners."

London: BAILLIÈRE, TINDALL & COX, 8 Henrietta St., Covent Garden.

---

### THE HANDBOOK OF THE OPEN-AIR TREATMENT

By Dr. CHARLES REINHARDT
(Visiting Physician to Hailey Sanatorium, Wallingford, and Stourfield Park Sanatorium, Bournemouth),

Contains Views, Descriptions, and Information respecting 30 Sanatoria in Great Britain.

Price: Cloth, 2/6; Paper, 1/- (postage 4d.)

The "HEALTH RESORT" Publishing Office, 379 Strand, London, W.C.

---

JUST OUT. With 3 coloured Plates, and 54 other Illustrations. Price 6/- net.

### HANDBOOK OF DISEASES OF THE EAR

For Students and Practitioners.

By RICHARD LAKE, F.R.C.S.Eng.,
Surgeon to the Royal Ear Hospital.

London: BAILLIÈRE, TINDALL & COX,
8 Henrietta Street, Covent Garden.

---

### CLINICAL LECTURES ON NEURASTHENIA.

By THOMAS D. SAVILL, M.D.Lond. Second Edition.

"Deals very exhaustively with the subject, and will be read with great interest."—*The Lancet.*

"It should be in the hands of every practitioner who wishes to keep himself well informed on this important subject."—*Treatment.*

H. J. GLAISHER, 57 Wigmore Street, W. 5/- net; 5/4 by post.

---

WORKS BY Mr. McADAM ECCLES, M.S., F.R.C.S.,
Assistant Surgeon St. Bartholomew's Hospital, &c.

### HERNIA.

SECOND EDITION, 1902. Price 7/6 net. Illustrated by Numerous Photographs.

### THE IMPERFECTLY DESCENDED TESTIS.

Just published. Profusely Illustrated. Price 7/6.

London: BAILLIÈRE, TINDALL & COX.

---

Crown 8vo., price 3/6.
With a "Plea for the State Registration of Nurses."

### MOCK NURSES OF THE LATEST FASHION.

By FREDERICK J. GANT, F.R.C.S.,
Consulting Surgeon to the Royal Free Hospital.

BAILLIÈRE, TINDALL & COX,
Henrietta Street, Covent Garden, London.

---

Crown 8vo. cloth, price 5s. net; by post, 5s. 4d.

*Second Edition of*

### DR. HEYWOOD SMITH'S PRACTICAL GYNÆCOLOGY.

This is a genuine handbook on the subject, intended for ready reference by busy practitioners.

W. J. GLAISHER, 57 Wigmore Street, Cavendish Square, W.

**Notices of Births, Deaths, and Marriages** are inserted in this column at a charge of 2s. 6d. for an announcement not exceeding four lines.

**DEATH.**

TURNER.—On October 31 at Duffield House, Bexhill-on-Sea, Nursing Sister A. E. TURNER, late of the Imperial Yeomanry Hospital, Pretoria, South Africa. (1997)

### NOTICE TO CORRESPONDENTS.

All MSS., Letters, Books for Review, and other matters intended for the Editor should be addressed THE EDITOR, "THE HOSPITAL," THE HOSPITAL BUILDINGS, 28 & 29 SOUTHAMPTON STREET, STRAND, W.C.

The Editor cannot undertake to return rejected MS., even when accompanied by stamped directed envelope.

**Notice to Advertisers and Subscribers.**

All Advertisements, Orders for Copies of THE HOSPITAL, and Business Communications should be addressed to **THE MANAGER (Not to the Editor)**, THE HOSPITAL, 28 & 29 Southampton Street, Strand, London, W.C.

The Cost of Subscription, post free, is as follows:—Per week, 3½d.; per quarter, 3s. 9d.; per half-year, 7s. 6d.; per annum, 15s. Foreign and Colonial:—Per annum, 19s. 6d., payable, in all cases, in advance.

**NOTICE.—Vol. XXXIV. of "The Hospital" (April 1903 to September 1903), in handsome cloth binding, is now on sale at the office of this Journal, price 7s. 6d. Cases for binding the Numbers contained in this Volume 1s. 6d. Index to Vol. XXXIV., 2½d., post free.**

**The Monthly part for November is now ready. Price 1s., by post, 1s. 3d.**

### BOOKS RECEIVED.

H. J. GLAISHER.

"Ethyl Chloride." By A. De Prenderville.

W. TYLER.

"The Art of Photographic Dodging." By W. Tyler.

SCOTT PUBLISHING COMPANY.

"Morals: the Psychosocialogical Bases of Ethics." By G. L. Duprat.

ELLIOT STOCK.

"The Home Nurse." By S. T. A. Caulfield.

BEMROSE AND SON.

"Beautiful Biskra." By C. Howard Tripp.

J. B. LIPPINCOTT AND CO.

"A Nurses' Handbook of Obstetrics." By J. B. Cooke, M.D.

"International Clinics." Vol. III.

"Principles and Practice of Surgery." By G. T. Vaughan.

SIMPKIN, MARSHALL AND CO.

"Essays and Notes on Hydrotherapeutics." By R. Metcalfe.

"Natural Physical Remedies." By H. H. Herbert.

THE SCIENTIFIC PRESS, LIMITED.

"A Case Book for Private Nursing."

T. NELSON AND SONS.

"Animal Physiology Diagrams."

### SCRAPS AND GLEANINGS.

ONE hundred and seventy members of Parliament have sent in their names as being desirous of taking part in the M.P.'s forthcoming visit to Paris.

A FURTHER sum of 1,000 guineas has been collected and handed to the Infirmary Committee for the endowment of a bed in the new ward for women in the Cardiff Infirmary.

THE Cuckfield Isolation Hospital Committee have had under consideration the question as to whether a charge could be made to patients who could afford to pay for their maintenance and who were not ratepayers, the hospital having been built and being maintained at the ratepayers' expense for the reception of patients free of charge. In view, however, of the desirability, in the interests of the public, of cases of infectious disease being isolated as quickly and efficaciously as possible, and of the difficulties which would be likely to arise if non-ratepayers who could afford to pay were charged for their treatment at the hospital, it has been decided by the committee that no charge shall be made for the admission of patients from parishes in the rural district, but that any patients wishing to pay for their maintenance may be allowed to do so.

### THE STUDENT OF MEDICINE.

### APPOINTMENTS.

**W. J. Bacque**, M.R.C.S.Eng., L.R.C.P.Lond., District Medical Officer of the Bath Union. **Robert Cozens Bailey**, M.S.Lond., F.R.C.S., Assistant Surgeon to St. Bartholomew's Hospital. **Sidney C. H. Bent**, M.D.Brux., M.R.C.S., L.R.C.P.Lond., Senior House Surgeon to the Evelina Hospital for Sick Children, Southwark. **Robert J. Blackham**, L.R.C.P.E., Captain R.A.M.C., Clinical Assistant in the London School of Gynæcology, the Hospital for Women, Soho Square, W. **Eleanor E. Bourne**, M.B., Ch.M.Syd., Resident Medical Officer to the Brisbane General Hospital. **J. J. Browne**, M.R C.S., L.R.C.P., Certifying Factory Surgeon for the Wigton District, Cumberland. **Frederick Irving De Lisle**, L.R.C.P.Edin., L.S.A., D.P.H., District Medical Officer, Wellington, New Zealand. **William Henry Duncan**, L.R.C.P. and L.R.C.S.Edin., House Surgeon to Nottingham Children's Hospital. **A. G. Ede**, M.B.Lond., District Medical Officer of the Ongar Union. **E. W. Fairfax**, M.B., Ch.M.Syd. M.R.C.S. Eng., Honorary Anæsthetist to the University Dental Hospital, Sydney, N.S W. **W. T. Farncombe**, M.D.Brux., M.R.C.S., L.R.C.P.Lond., Certifying Factory Surgeon for the Selly Oak District Worcestershire. **Alfred Greenwood**, M.D., D.P.H., Medical Officer to the Blackburn Education Authority. **A. H. Heslop**, M.B. B.S.Durh., Second Assistant Medical Officer of the City Asylum, Gosforth. **Alexander Lyons**, M.R C.P., L.R.C.S. Edin., House Surgeon to the Royal Eye Hospital Southwark. **E. D. Parsons**, M.R.C.S., L.R.C.P.Lond., House Physician to the Evelina Hospital for Sick Children, Southwark. **W. Penberthy**, M.R.C.S., L.R.C.P.Lond., Certifying Factory Surgeon for the Wiveliscombe District, Somerset. **William Stuart Low**, F.R.C.S.Eng., Assistant Surgeon to the Central Throat and Ear Hospital, Gray's Inn Road. **W. J. Symes**, M.B., B.S.Durh., Medical Officer and Public Vaccinator for the Chesterfield District of the Chesterfield Union. **Charles E. Thomas**, M.R.C.S.Eng., L.S.A., Port Health Officer for the Port of Timaru, New Zealand. **E. R. Wheeler**, M.R.C.S., L.R.C.P.Lond., Assistant House Surgeon to the Evelina Hospital for Sick Children, Southwark. **R. H. Anglin Whitelocke**, M.D., M.Ch.Edin., F.R.C.S.Eng., Lichfield Lecturer in Clinical Surgery in the University of Oxford.

**"The Hospital" Institutional Library.**

**"Hospitals and Asylums of the World." 4 Vols., with a Portfolio of Plans, complete £12 12s.**
**"Burdett's Hospitals and Charities, 1903." 5s. net.**
**"Burdett's Official Nursing Directory, 1903." 3s. net.**
**"Cottage Hospitals, General, Fever, and Convalescent." 10s. 6d.**
**"Uniform System of Accounts for Hospitals and Public Institutions." New Edition. 4s. net.**
**"Hospital Expenditure: The Commissariat." 2s. 6d.**
**"A Legal Handbook for the Use of Hospital Authorities." 2s. 6d.**
**"The Cottage Hospital Case Book or Register of Patients." 10s. and 12s. 6d.**

**All these are published by the SCIENTIFIC PRESS, Ltd., and may be obtained through any bookseller, or direct from the publishers, 28 and 29 Southampton Street, Strand, London, W.C.**

**MALTICO** For **INFANTS** and **INVALIDS.**
ALL BABIES LIKE & THRIVE ON MALTICO.
*Send for Samples, Analytical Reports and all particulars.*
**MALTICO FOOD COMPANY, Kingston Cross, Portsmouth.**

**MEDICAL HISTORY FROM THE EARLIEST TIMES.**

By E. T. WITHINGTON, M.A., M.B. Oxon.

Demy 8vo., over 400 pp., with two Plates, cloth gilt, **12/6 net.**

"One of the best attempts that has yet been made in the English language to present the reader with a concise epitome of the history of medicine, and as such we very cordially commend it."

*Glasgow Medical Journal.*

THE SCIENTIFIC PRESS, LTD.,

28 and 29 Southampton Street, Strand, London, W.C.

THE NURSING PROFESSION: How and Where to Train.
By Sir HENRY BURDETT, K.C.B.
A reliable and up-to-date Guide to Training for the calling of a Nurse. In its pages would-be Nurses will find all the information they require.
New Edition. Price 2/-, post free 2/4.
Of all Booksellers or of THE SCIENTIFIC PRESS, LTD., 28 & 29 Southampton Street, Strand, London, W.C.

**ON PREPARATION FOR OPERATION IN PRIVATE HOUSES.**

By STANMORE BISHOP, F.R.C.S.

Crown 8vo. paper cover, **6d.**

THE SCIENTIFIC PRESS, LIMITED,
28 & 29 Southampton Street, Strand, London, W.C.

### Important to Nurses.

***Just Published.***

### THE EXAMINATION OF THE URINE.

**By J. K. WATSON, M.D. Edin., M.B., C.M.,**

Author of a "Handbook for Nurses."

*CONTENTS:—*

**PART I.**

**Preliminary—The Urinary Apparatus.**

**PART II.**

**The Urine. Its Physical Characters in Health and Disease.**

**PART III.**

**The Collection and Examination of the Urine.**

Suitable size for the Apron Pocket. Bound in cloth boards, **1/-** net; post free **1/1.**

### A NEW CASE BOOK FOR PRIVATE NURSING.

**Arranged for Day and Night Nurse's Report, with rulings in accordance with what has been found in practice to be the most suitable and approved System for Pay Hospitals, Nursing Homes, Private Nurses, &c.**

DAY NURSE'S REPORT.

*Date.............*

| Time | Nourishment | Quantity | Urine | Stimulants, Medicines, and Remarks |
|---|---|---|---|---|
| | | | | |

| Total Nourishment | Bowels | Summary |
|---|---|---|
| | | |

(Facsimile of ruling.)

**Price 3d. net.** Post free $4\frac{1}{2}$d.

**The Size of an Exercise Book ($7\frac{1}{2}$ in. by $9\frac{1}{2}$ in.).**

**Price 3d. net.** Post free $4\frac{1}{2}$d.

*Obtainable of all Booksellers.*

THE SCIENTIFIC PRESS, LIMITED, 28 & 29 Southampton Street, Strand, London, W.C.

Standard Works

### J. & A. CHURCHILL'S Books for Nurses.

Latest Editions

### NURSING.

Nursing, General, Medical and Surgical, with an Appendix on Sick Room Cookery. By WILFRED J. HADLEY, M.D., F.R.C.P., F.R.C.S., Physician and Pathologist to the London Hospital, Lecturer to Nurses at the London Hospital Nursing School . . . . . 3s. 6d.

### MEDICINE.

Lectures on Medicine to Nurses. By HERBERT E. CUFF, M.D., F.R.C.S., Medical Superintendent, North Eastern Fever Hospital, London. Fourth Edition. With 29 Illustrations . . . . . 3s. 6d.

### INFANT FEEDING.

The Natural and Artificial Methods of Feeding Infants and Young Children. By EDMUND CAUTLEY, M.D., F.R.C.P., Physician to the Belgrave Hospital for Children; late Casualty Physician and Assistant Demonstrator of Physiology, St. Bartholomew's Hospital. Second Edition. 7s. 6d.

### DIET.

Diet and Food considered in relation to Strength and Power of Endurance, Training and Athletics. By A. HAIG, M.D., F.R.C.P. Fourth Edition. 2s.

### MIDWIFERY.

A Short Practice of Midwifery for Nurses. By HENRY JELLETT, M.D. (Dub. Univ.), F.R.C.P.I., Ex-Assistant Master, Rotunda Hospital, Dublin, Extra Examiner in Midwifery and Gynæcology, Royal College of Physicians of Ireland, Extern Examiner in Midwifery, Royal University of Ireland. With 67 Illustrations . . . . 6s.

### MONTHLY NURSING.

A Short Manual for Monthly Nurses. By C. J. CULLINGWORTH, M.D., F.R.C.P., Obstetric Physician to St. Thomas's Hospital. Revised with the assistance of M. A. ATKINSON, Matron of the General Lying-in Hospital, London. Fifth Edition. 1s. 6d.

### HEALTH OF A WIFE.

Chavasse's Advice to a Wife on the Management of her Own Health. Fourteenth Edition. By FANCOURT BARNES, M.D., Consulting Physician to the British Lying-in Hospital . . . . . 2s. 6d.

### HEALTH OF CHILDREN.

Chavasse's Advice to a Mother on the Management of her Children. Fifteenth Edition. By GEORGE CARPENTER, M.D., Physician to the Evelina Hospital for Children . . . . . 2s. 6d.

### ANTISEPTICS.

Lessons in Disinfection and Sterilisation; an Elementary Course of Bacteriology, with a Scheme of Practical Experiments. By F. W. ANDREWES, M.D., F.R.C.P., Lecturer on Pathology at St. Bartholomew's Hospital. With Illustrations . 3s. net. [*Just Published.*

### SURGERY.

A Manual of Minor Surgery and Bandaging. By CHRISTOPHER HEATH, F.R.C.S., and BILTON POLLARD, F.R.C.S., Surgeon to University College Hospital. Twelfth Edition. With 195 Illustrations. 6s. 6d.

### HYGIENE.

The Elements of Health: An Introduction to the Study of Hygiene. By LOUIS C. PARKES, M.D., D.P.H.Lond., Lecturer on Public Health at St. George's Hospital. 3s. 6d.

### DICTIONARY.

A Short Dictionary of Medical Terms. By R. G. MAYNE, M.D., LL.D. . 2s. 6d.

7 GREAT MARLBOROUGH STREET, LONDON.

